# Phospholipase A_2_ products influence the antiplatelet functions of synthetic high-density lipoproteins

**DOI:** 10.1016/j.jlr.2025.100972

**Published:** 2025-12-29

**Authors:** Antonela Rodriguez, Minzhi Yu, Jingyao Gan, May Thazin Phoo, Alankrita Rani, Gunther Marsche, Yanhong Guo, Michael Holinstat, Anna Schwendeman

**Affiliations:** 1 Department of Pharmaceutical Sciences, University of Michigan, Ann Arbor, MI, USA; 2 Biointerfaces Institute, University of Michigan, Ann Arbor, MI, USA; 3 Division of Pharmacology, Otto Loewi Research Center, Medical University of Graz, Graz, Austria; 4 Department of Internal Medicine, University of Michigan, Ann Arbor, MI, USA; 5 Department of Pharmacology, University of Michigan, Ann Arbor, MI, USA

**Keywords:** synthetic high-density lipoprotein, lipoproteins/metabolism, platelets, phospholipases/A2, lysophospholipid, DMPC, lipids, apolipoprotein A1 mimetic peptides

## Abstract

Multiple synthetic high-density lipoproteins (sHDLs) have been developed and extensively evaluated in preclinical and clinical trials, with their functionality potentially linked to specific lipid compositions. This study investigates how lipid composition influences sHDL interactions with platelets. We synthesized sHDL particles using ApoA1 mimetic peptide 22A complexed with various lipids (DMPC, POPC, DSPC, DPPC, and SM) differing in chain lengths, saturation levels, and transition temperatures. DMPC sHDL demonstrated superior inhibition of platelet aggregation across multiple agonist concentrations, while POPC sHDL showed limited efficacy only at lower thrombin concentrations. Interestingly, all formulations exhibited similar cholesterol removal abilities, and POPC sHDL demonstrated the highest platelet association despite its inferior antiplatelet effects. Mechanistic investigation revealed the involvement of phospholipase A_2_ (PLA_2_) enzymes in DMPC sHDL’s potent antiplatelet effects. Inhibition of cytosolic PLA_2_ (cPLA_2_) and lipoprotein-associated PLA_2_ (Lp-PLA_2_) significantly reduced DMPC sHDL's antiplatelet activity. We demonstrated PLA_2_-mediated hydrolysis of DMPC sHDL, resulting in bioactive lipid metabolites, lysophosphatidylcholine (LPC) 14:0 and myristic acid, both in vitro and in vivo. These metabolites directly inhibited platelet aggregation, integrin activation, and α-granule secretion in a dose-dependent manner, with significantly greater potency than metabolites derived from other phospholipids. Our findings elucidate a novel mechanism by which sHDL’s lipid composition influences its antiplatelet properties through the generation of bioactive lipid metabolites, offering insights for developing targeted cardiovascular therapies.

High-density lipoproteins (HDLs) play a crucial role in cardiovascular health, exhibiting a wide range of biological activities beyond their well-known function in reverse cholesterol transport (1, 2, 3, 4). These activities include anti-inflammatory, antioxidant, and antithrombotic properties, all contributing to HDL’s cardioprotective effects. Given the multifunctionality of these endogenous particles, several HDL-mimicking therapies, known as synthetic HDLs (sHDLs), have been developed and investigated for cardiovascular disease (5).

Several sHDL formulations have progressed through various phases of clinical trials targeting atherosclerosis complications, including CSL-111, CSL-112, ETC-216, and CER-001 (5). Despite initial promise, many therapies have been discontinued or failed to meet primary endpoints, as evidenced by recent reports on CSL-112 phase III trial (6, 7). These sHDLs were primarily designed to enhance cholesterol efflux from atherosclerosis plaques (8, 9), while other bioactive properties remained largely underexplored. Previous observations have suggested that the lipid content of native HDLs is closely linked to their functional properties (3, 4, 10). Current sHDL therapies consist of plasma-purified apolipoprotein A1 (ApoA1) or recombinant ApoA1 complexed with lipids such as soybean phosphatidylcholine, 1-palmitoyl-2-oleoyl-sn-glycero-3-phosphocholine (POPC), dipalmitoylphosphatidyl glycerol (DPPG), and sphingomyelin (SM). Therefore, recent clinical failures may stem from formulations designed solely to enhance cholesterol efflux, with limited consideration of how lipid composition affects other bioactive properties.

To address these limitations, our group has previously explored the anti-inflammatory properties of sHDLs and the effects of lipid composition effects on their activity. Our studies demonstrate that lipid content dictates both the cholesterol efflux capacity (11, 12, 13) and anti-inflammatory actions (12, 13, 14) of sHDLs. Recognizing thrombosis as a critical factor in cardiovascular disease, we expanded our investigations to examine the antithrombotic properties of sHDLs through their interactions with platelets. Platelets are central mediators in both thrombosis and atherosclerosis progression, making their effective regulation essential for preventing atherothrombotic events (2, 15, 16). Our findings revealed that sHDL effects on platelet functions depend on both the lipid and protein components, with 1,2-dimyristoyl-sn-glycero-3-phosphocholine (DMPC)-based sHDLs exhibiting greater capacity to inhibit platelet reactivity compared to DMPC micelles (17). Furthermore, we showed that these DMPC sHDLs modulate various platelet functions in vitro including aggregation, integrin activation, α-granule secretion, protein kinase C (PKC) activity, and platelet spreading (17, 18). We determined that sHDL’s antithrombotic functions were not exclusively dependent on cholesterol efflux and were mediated through CD36 engagement. Importantly, in in vivo thrombosis models, DMPC sHDL inhibited thrombus formation and delayed vessel occlusion time (18).

Expanding on these observations, the present study systematically investigates whether lipid composition similarly influences the antiplatelet properties of sHDL. We designed a series of sHDLs formulations incorporating lipids with distinct physical properties, including varying chain lengths, saturation levels, and transition temperatures. We synthesized sHDL particles using ApoA1 mimetic peptide 22A complexed with various lipids: DMPC, POPC, 1,2- distearoyl-sn-glycero-3-phosphocholine (DSPC), 1,2-dipalmitoyl-sn-glycero-3-phosphocholine (DPPC), and SM. We then evaluated the sHDL formulations on the antiplatelet activity, cholesterol removal capacity, and cellular association.

Our findings reveal that DMPC sHDL inhibited platelet aggregation to a greater extent than other sHDLs. Although all sHDL particles removed similar levels of total cholesterol, they had differences in platelet association, with POPC sHDL exhibiting highest level of interactions with platelets. These results indicate that the antiplatelet activity of sHDLs is not solely determined by their cholesterol removal capacity or degree of cellular association. To further understand the mechanisms underlying DMPC sHDL’s potent antiplatelet effects, we investigated the probable role of phospholipase A_2_ (PLA_2_) enzymes. We demonstrate that inhibition of PLA_2_, specifically cytosolic PLA_2_ (cPLA_2_) and lipoprotein-associated PLA_2_ (Lp-PLA_2_), significantly reduces DMPC sHDL’s antiplatelet activity. Furthermore, we show that PLA_2_ hydrolyze DMPC in sHDL, resulting in the production of bioactive lipid metabolites – LPC 14:0 and myristic acid – both in vitro and in vivo. Notably, these metabolites directly modulate platelet functions in a dose-dependent manner. This work reveals a novel mechanism underlying the unique antiplatelet properties of DMPC-based sHDL and highlights the importance of lipid composition in determining sHDL’s therapeutic potential for cardiovascular disease.

## Materials and Methods

### Materials

ApoA1 mimetic peptide 22A (PVLDLFRELLNELLEALKQKLK) was synthesized by GenScript Inc. (Piscataway, NJ). Lipids: 1,2-dimyristoyl-sn-glycero-3-phosphocholine (DMPC), 1-palmitoyl-2-oleoyl-sn-glycero-3-phosphocholine (POPC), 1,2- distearoyl-sn-glycero-3-phosphocholine (DSPC), 1,2-dipalmitoyl-sn-glycero-3-phosphocholine (DPPC), and sphingomyelin (SM) were purchased from NOF America Corporation (White Plains, NY); 1-myristoyl-2-hydroxy-sn-glycero-3-phosphocholine (LPC 14:0), 1-tridecanoyl-sn-glycero-3-phosphocholine (LPC 13:0), and 1,2-dimyristoyl-d54-sn-glycero-3-phosphocholine (PC-d54 14:0) were purchased from Avanti Polar Lipids (Alabaster, AL); and myristic acid and tridecanoic acid were purchased from Sigma-Aldrich (St. Louis, MO). Additional lipids: 2-thio PAF (1-O-hexadecyl-2-deoxy-2-thio-S-acetyl-*sn*-glyceryl-3-phosphorylcholine), PAF C-18 (1-O-octadecyl-2-O-acetyl-*sn*-glyceryl-3-phosphorylcholine), and Lyso-PAF C-18 (1-O-octadecyl-*sn*-glyceryl-3-phosphorylcholine) were purchased from Cayman Chemical (Ann Arbor, MI). Phospholipase inhibitors AACOCF3 and darapladib from Tocris Bioscience (Bristol, UK), varespladib (LY315920) from Selleck Chemicals (Houston, TX), bromoenol lactone (BEL) from Cayman Chemical (Ann Arbor, MI), and FIPI from Sigma-Aldrich (St. Louis, MO). Thrombin was purchased from Enzyme research laboratories (South Bend, IN), collagen from Chrono-Log (Havertown, PA), and convulxin from Kenneth Clemetson (Theodor Kocher Institute, University of Berne, Bern, Switzerland). All reagents used in this study were reagent grade or higher.

### Preparation and characterization of sHDL nanoparticles

sHDL containing ApoA1 mimetic peptide 22A and various phospholipids (DMPC, POPC, DSPC, DPPC, and SM) were prepared using the previously developed lyophilization method (11). Briefly, 22A peptide and corresponding phospholipids were dissolved in glacial acetic acid at a 1:2 (peptide:lipid) w/w ratio, followed by lyophilization for 24 h. The resulting lyophilized powder was then rehydrated in 1X PBS and subjected to three thermal cycles. Each cycle involved heating to ∼15°C above the phospholipid’s transition temperature (T_m_), followed by cooling below T_m_ for 5 min each. After thermal cycling, the solution’s pH was adjusted to 7.4. All formulations were purified using a Zeba Spin Desalting Column (2 ml, ThermoFisher Scientific, Waltham, MA) and 0.22 μm syringe filter. The particle size was determined using dynamic light scattering (DLS) on Malvern Zetasizer Nano ZSP (Westborough, MA). Purity of the particles was confirmed by gel permeation chromatography (GPC) on a Tosoh TSK gel G3000SWxl column (Tosoh Bioscience, King of Prussia, PA) with PBS as mobile phase with flow rate of 1 ml/min and UV detection at 220 nm. The particle morphology was observed by transmission electron microscopy (TEM). For TEM analysis, samples were loaded on a carbon film-coated 400-mesh copper grid (Electron Microscopy Sciences; Hatfield, PA), negatively stained with 1% (w/v) uranyl formate, and dried. The final concentrations of 22A peptide and lipids were quantified using Waters® ACQUITY UPLC H-Class system equipped with a QDa detector (Waters, Milford, MA). Chromatographic separation was performed on a Waters® ACQUITY UPLC® Protein BEH C4 (1.7 μm, 2.1 x 100 mm) column. Mobile phase A consisted of water and mobile phase B was 50:50 acetonitrile:methanol, both containing 0.1% formic acid. Separation was carried out at a flow rate of 0.3 ml/min and at a column temperature of 50°C, using the following gradient: initial (15% B), 0–6 min (100% B), 6–9 min (hold 100% B), 9–9.5 min (7% B), 9.5–15.5 min (hold 7% B). Samples were injected at a volume of 1 μl. Selected ion recording (SIR) in positive ion mode with cone voltage of 15V was used to detect: 22A (m/z 657.0), DMPC (m/z 678.5), POPC (m/z 760.6), DSPC (m/z 790.6), DPPC (m/z 734.6), and SM (m/z 703.6).

### Washed human platelet preparation

All human subject research complied with the Declaration of Helsinki and was conducted under the approval from the University of Michigan Institutional Review Board (HUM00100677). Written informed consent was obtained from all healthy donors prior to blood collection. Blood samples were collected in sodium citrate-containing vacutainers and centrifuged at 200g for 10 min. Platelet-rich plasma (PRP) was separated and mixed with acid citrate dextrose (2.5% sodium citrate tribasic, 1.5% citric acid, 2.0% d-glucose) and apyrase (0.02 U/ml). Platelets were isolated by further centrifugation (2000g, 10 min) and resuspended in Tyrode’s buffer (10 mM HEPES, 12 mM sodium bicarbonate, 127 mM sodium chloride, 5 mM potassium chloride, 0.5 mM monosodium phosphate, 1 mM magnesium chloride, and 5 mM glucose) to a final concentration of 3.0 x 10^8^ platelets/ml. No albumin was supplemented during platelet resuspension, consistent with previously established protocols (17, 18).

### Platelet aggregation

Washed platelets (3.0 x 10^8^ platelets/ml) were subjected to various treatments at specified times and concentrations. Incubations were performed under stirring conditions (1100 rpm) at 37°C. Control groups included untreated platelets and/or platelets treated with inhibitors for comparison. Following treatments, aggregation was induced using thrombin or collagen and recorded for 10 min using a Chrono-log Model 700D Lumi-aggregometer (Havertown, PA).

For platelet aggregation in PRP, PRP was isolated from citrated whole blood by centrifugation (200g for 10 min). Platelet-poor plasma was used to adjust PRP platelet counts to 3.0 x 10^8^ platelets/ml. PRP was incubated with treatments under stirring conditions (1100 rpm at 37°C), untreated PRP was used as control. Prior to inducing aggregation, PRP was recalcified by adding 5 μl of 100 mM CaCl_2_ and incubated for 3 min. Aggregation was then induced using collagen or ADP and recorded for 10 min.

### Quantification of total cholesterol in platelets

Washed platelets (3.0 x 10^8^ platelets/ml) were treated with all sHDL formulations at approximately 100 μg/ml of 22A peptide concentration (corresponding to about 200 μg/ml of lipid), or 22A peptide (100 μg/ml) for 15 min at 37°C. Following incubation, platelets were treated with acid citrate dextrose and apyrase and centrifuged at 1000g for 10 min, and the pellet was resuspended in Tyrode’s buffer at 3.0 x 10^8^ platelets/ml. Untreated platelets were used as controls, the same procedure was followed. The total cholesterol was determined using the Amplex Red Cholesterol Assay Kit (Invitrogen, ThermoFisher Scientific, Waltham, MA) according to the manufacturer’s procedures.

### Quantification of sHDL association with platelets

To evaluate the association of various sHDL formulations with platelets, DiI (Thermo Fisher Scientific, Waltham, MA) was incorporated into sHDL, as previously described (17, 18). DiI-labeled sHDL was used to quantify association with platelets. Note that this approach measures overall interaction between sHDL and platelets rather than demonstrating complete particle internalization. Briefly, 22A peptide, lipids, and DiI were dissolved in glacial acetic acid at 1:2:0.01 (22A:DMPC:DiI) weight ratio. The mixture was freeze-dried overnight, rehydrated in 1X PBS, thermally cycled (3x), and final pH was adjusted to 7.4. The final formulations were filtered using a 0.22 μm syringe filter. Washed platelets (3.0 x 10^8^ platelets/ml) were incubated with the DiI-labeled sHDL formulations (approximately 100 μg/ml 22A, 200 μg/ml lipid, and 1 μg/ml DiI) for 5, 15, 30, and 60 min and subsequently fixed with 2% paraformaldehyde in Tyrode’s buffer to a final concentration of 6.0 x 10^7^ platelets/ml. The mean fluorescence intensity (MFI) of DiI content in platelets was quantified by flow cytometry on a Beckman Coulter Cytoflex Flow Cytometer (Indianapolis, IN).

### Quantification of PLA_2_ hydrolysis products via LC/MS

#### Sample preparation

Washed platelets (3.0 x 10^8^ platelets/ml) were incubated with 22A:DMPC sHDL (approximately 22A 100 μg/ml and DMPC 200 μg/ml) for 15 min under stirring conditions at 37°C. Then, platelets were activated with thrombin or collagen for 5 min. Untreated platelets, both unstimulated and stimulated, were included as controls. For quantification of hydrolysis products for sHDLs containing different lipids, platelets were incubated with 22A:POPC sHDL, 22A:DPPC sHDL, and 22A:DSPC sHDL (approximately 100 μg/ml 22A and 200 μg/ml lipid) for 15 min under stirring at 37°C. Platelets were centrifuged at 1000g for 3 min, and both pellet and supernatant were collected. Samples were flash-frozen in liquid nitrogen and stored at −80°C.

All protocols involving mice in this study were approved by the Institutional Animal Care & Use Committee (IACUC) at the University of Michigan, Ann Arbor. For the in vivo study, male mice (C57BL/6J) received either vehicle or DMPC sHDL (100 mg/kg peptide and 200 mg/kg DMPC) via intravenous injection. Blood samples were collected from the submandibular vein in heparinized tubes at 0.25, 2, 4, 8, and 24 h post-injection. Plasma was obtained by centrifugation (5000 rpm for 10 min at 4°C) and stored at −80°C.

Lipid extraction was performed on both pellet and supernatant using a modified Bligh-Dyer method (19). For the cell pellet, a 1:1:2 ratio of water:methanol:chloroform was used, while for the supernatant, a 1:2 ratio of methanol:chloroform was used. Internal standards were added to all samples prior to extraction: LPC 13:0 (for LPC 14:0), tridecanoic acid (for myristic acid), and PC-d54 14:0 (for DMPC). Similarly, for the samples containing different sHDLs, we utilized LPC 13:0 and tridecanoic acid as internal standards for the LPCs and fatty acids, respectively. For plasma samples, lipid extraction was done by adding 1:1:2 ratio of water:methanol:chloroform. Internal standards were added as before. Samples were vortexed for 3 min and centrifuged at 4000 g. The organic layer was collected, dried under nitrogen, and reconstituted in mobile phase B (76:19:5 methanol:acetonitrile:isopropanol with 5 mM ammonium acetate).

#### Targeted LC/MS analysis

DMPC hydrolysis products were quantified using Waters® ACQUITY UPLC H-Class system equipped with a QDa detector (Waters, Milford, MA). Chromatographic separation was performed on a Waters® ACQUITY UPLC® BEH C18 (1.7 μm, 2.1 x 50 mm) column, at a constant column temperature of 50°C. Mobile phase A consisted of 20:80 methanol:water, while mobile phase B was 76:19:5 methanol:acetonitrile:isopropanol, both containing 5 mM ammonium acetate. Separation was carried out at a flow rate of 0.5 ml/min with a 10-min gradient: initial (30% B), 0–2 min (70% B), 2–4 min (80% B), 4–7 min (99% B), 7–8 min (hold 99% B), 8–8.1 min (30% B), 8.1–10 min (hold 30% B). Samples and standards were injected at a volume of 2 μl. SIR was used to detect lipid species of interest at a cone voltage of 15V. Positive mode was used for LPC 14:0 (m/z 468.3), LPC 16:0 (m/z 496.3), LPC 18:0 (m/z 524.3), and DMPC (m/z 678.5), as well as internal standards LPC 13:0 (m/z 454.3) and PC-d54 14:0 (m/z 732.8). Negative mode was used for myristic acid (m/z 227.0), oleic acid (m/z 281.4), palmitic acid (m/z 255.2), stearic acid (m/z 283.3), and internal standard tridecanoic acid (m/z 213.2).

### Hydrolysis of DMPC via Lp-PLA_2_

Recombinant human Lp-PLA_2_ protein (R&D Systems; Minneapolis, MN) was used to investigate the direct hydrolysis of DMPC. First, enzyme activity was validated using a colorimetric assay with 2-thio PAF substrate according to manufacturer’s instructions with modifications. Briefly, recombinant Lp-PLA_2_ (2.5 μg/ml) was incubated with varying concentrations of 2-thio PAF (25, 50, 100, 200, and 400 μM) in assay buffer (50 mM MES, 150 mM NaCl, 0.1 mg/ml BSA, pH 6.5) along with 5,5′-dithio-bis(2-nitrobenzoic acid) (DTNB; Sigma-Aldrich, St. Louis, MO) at 37°C. Enzymatic hydrolysis of 2-thio PAF was monitored by measuring the release of free thiol groups using DTNB at 405 nM with a microplate reader. Absorbance readings were recorded every minute for 10 min, and the initial velocity (Abs/min) was calculated from the linear portion of the progress curve. To confirm inhibitor efficacy, the assay was repeated with 2-thio PAF (400 μM) in the presence or absence of Lp-PLA_2_-specific inhibitor darapladib (0.5 μM). Prior to incubation with PAF, Lp-PLA_2_ was incubated with darapladib for 10 min. Absorbance values were recorded over time and plotted to visualize inhibition.

For LC-MS analysis of enzymatic activity, both established Lp-PLA_2_ substrates and DMPC were tested. First, 2-thio PAF (400 μM) and PAF C18 (400 μM) were incubated with recombinant Lp-PLA_2_ (2.5 μg/ml) in assay buffer at 37°C for 15 min in the presence or absence of darapladib (0.5 μM). Following incubation, samples were placed in ice and processed for lipid extraction and LC-MS analysis according to the previous section (*Quantification of DMPC PLA*_*2*_ *hydrolysis products* via *LC/MS*). Lipids were detected via SIR in positive ion mode: Lyso-2-thio PAF (m/z 498.4) and LysoPAF C18 (m/z 510.4). For PAF C18, the percentage increase in LysoPAF C18 was calculated by comparing levels in enzyme-treated samples relative to control samples without enzyme.

To assess DMPC hydrolysis, DMPC at various concentrations (200, 400, 800 μM) was incubated with recombinant Lp-PLA_2_ (2.5 μg/ml) in assay buffer for 15 min at 37°C. To determine potential differences in hydrolysis between free DMPC and DMPC incorporated in sHDL, and to further confirm the effects of darapladib, both DMPC alone and DMPC sHDL (containing equivalent DMPC concentrations of 400 μM) were incubated with Lp-PLA_2_ for 15 min at 37°C in the presence and absence of darapladib (0.5 μM). All samples were processed using the same lipid extraction and LC-MS analysis method as before. The percentage increases in LPC 14:0 and myristic acid were calculated by comparing levels in enzyme-treated samples relative to control samples without enzyme addition.

### Integrin αIIbβ3 activation and P-selectin surface expression

Washed platelets (3.0 x 10^8^ platelets/ml) were incubated with control, LPC 14:0, and myristic acid at concentrations of 1, 5, 10, 25, and 50 μg/ml at 37°C. Solvent used to dissolve lipids was used as control. Following treatments, 50 μl of platelets were incubated with thrombin (0.5 nM) or convulxin (25 ng/ml), along with antibodies anti-human PAC-1 FITC (BD biosciences, Franklin Lakes, NJ) and anti-human CD62P APC/Cyanine 7 (Biolegend, San Diego, CA) for 10 min. Platelets were fixed with 2% paraformaldehyde in Tyrode’s buffer to a final concentration of 6.0 x 10^7^ platelets/ml. Integrin αIIbβ3 activation and P-selectin surface expression were measured by flow cytometry on a Beckman Coulter Cytoflex Flow Cytometer. Percent (%) positive cells of PAC1 or CD62P were quantified.

### Statistical analysis

One-way analysis of variance (ANOVA) with Tukey’s multiple comparisons test and unpaired two-tailed t-tests were performed to compare experimental groups using Prism 10 (GraphPad Software). Data represents mean values ± standard deviation (SD). Significance was determined based on *∗P* < 0.05, *∗∗P* < 0.01, *∗∗∗P* < 0.001, *∗∗∗∗P* < 0.0001 or *#P* < 0.05, *##P* < 0.01, *###P* < 0.001, *####P* < 0.0001 or §*P* < 0.05, §§*P* < 0.01, §§§*P* < 0.001, §§§§*P* < 0.0001, as indicated in figure legends.

## Results

### Preparation and characterization of various sHDL particles

To investigate the effects of the lipid composition of sHDL on its interactions with platelets, we synthesized sHDL with 22A ApoA1 mimetic peptide combined with various lipids: DMPC, POPC, DSPC, DPPC, and SM. We selected these lipids based on their relevance to therapeutic sHDL development. POPC has been utilized in clinical HDL therapeutics such as ETC-216, while combinations including DPPC and SM have been employed in ETC-642 (5). DMPC was included based on our previous findings demonstrating its potent antithrombotic properties (17, 18). By systematically investigating lipids with varying chain lengths, saturation levels, and transition temperatures, we aimed to elucidate structure-activity relationships.

**Fig. 1:**
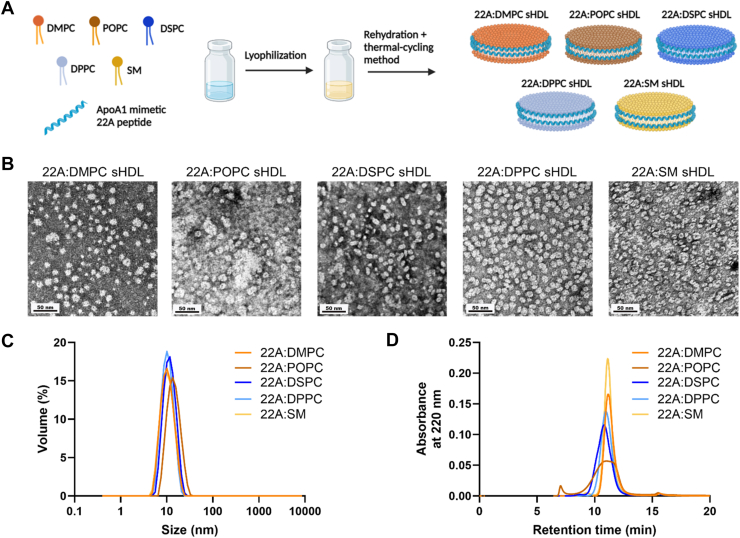
sHDL particles preparation and characterization. (A) Illustration of sHDL synthesis containing DMPC, POPC, DSPC, DPPC, or ESM, and 22A ApoA1 mimetic peptide (22A). Created with Biorender.com. (B) TEM images of sHDL particles; scale bar of 50 nm. (C) Particle size profile of sHDLs by DLS. (D) Purity and size distribution chromatograms of sHDLs by GPC.

All sHDLs were synthesized using the colyophilization method, combining peptide and lipids at a 1:2 w/w ratio (Fig. 1A) (11). The peptide-to-lipid ratios were further confirmed to be approximately 1:2 via LC-MS (Supplementary Table S1). This ratio was previously optimized to produce homogeneous sHDL nanoparticles resembling native discoidal preβ HDL (20). TEM was utilized to confirm the morphology of the nanoparticles (Fig. 1B), while DLS verified nanoparticle homogeneity and size distribution (Fig. 1C). All sHDLs demonstrated good homogeneity, with average hydrodynamic diameters ranging from 9.5 nm to 13.4 nm and low polydispersity index (<0.2). DMPC (9.5 nm), SM (9.6 nm), and DSPC (11.5 nm) showed the smallest sizes, while POPC (13.3 nm) and DPPC (13.4 nm) were larger. GPC analysis of sHDLs revealed similar retention times, with peaks eluting at approximately 11 min (Fig. 1D). POPC sHDL exhibited some heterogeneity, indicated by broader peak and presence of liposome impurities (∼7.5 min), however this impurity was very minimal accounting for less than 5%. All nanoparticles contained a negligible amount (<2%) of free 22A peptide.

### sHDL formulated with DMPC, but not other lipids, inhibits platelet aggregation

Our previous observations showed that the anti-inflammatory properties of sHDL were dependent on the lipid content (12, 13, 14). Similarly, native HDLs exhibit varying protective properties, particularly antithrombotic activities, depending on their lipid profiles (10, 21). We sought to evaluate whether the antiplatelet activities of sHDL can be affected by the lipid composition.

**Fig. 2:**
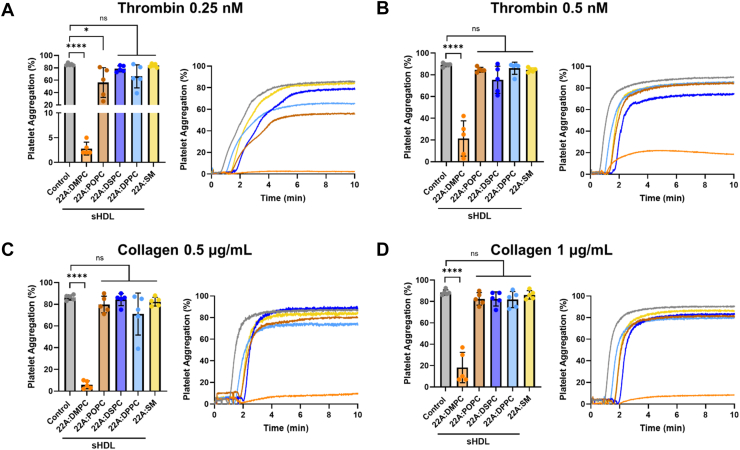
sHDL composed of DMPC phospholipid inhibits platelet aggregation. Washed human platelets were incubated with sHDL containing different lipid compositions for 15 min, with all sHDLs at a concentration of 100 µg/ml 22A peptide and 200 µg/ml lipid. Platelets were then stimulated with (A, B) thrombin (0.25 nM or 0.5 nM) or (C, D) collagen (0.5 µg/ml or 1 µg/ml) for 10 min. Data represent mean ± SD (n = 5), the maximum aggregation is displayed on the left and representative traces on the right; ns = not significant, *∗P* < 0.05, *∗∗∗∗P* < 0.0001.

We assessed the efficacy of sHDL formulations varying in lipid compositions at inhibiting platelet aggregation in response to two platelet agonists, thrombin and collagen, at both low and high concentrations. Platelets were treated with sHDLs at a uniform concentration of 100 μg/ml of the 22A peptide (200 μg/ml corresponding lipid concentration). When stimulated with a low concentration of thrombin (0.25 nM), both POPC and DMPC sHDLs significantly inhibited platelet aggregation (Fig. 2A). However, at higher thrombin concentrations (0.5 nM), only DMPC sHDL maintained potent inhibitory effects, while POPC sHDL lost its effectiveness (Fig. 2B). This pattern was also observed in response to collagen stimulation, where DMPC sHDL showed strong inhibition of aggregation across both concentrations, but POPC sHDL was not effective in either (Fig. 2C and D). The other formulations tested DSPC, DPPC, and SM sHDLs were not effective at inhibiting aggregation under any of the tested conditions (Fig. 2A–D). The inhibitory effects of DMPC sHDL on platelet aggregation were further evaluated in PRP to assess activity under more physiologically relevant conditions. DMPC sHDL significantly reduced aggregation in response to both collagen (1 μg/ml) and ADP (2.5 μM) compared to controls (Supplementary Fig. S1).

### Effect of phospholipid composition of sHDL on cholesterol removal capacity and platelet association

Given the varying inhibitory capacities of different sHDL formulations, particularly the potent inhibition by DMPC sHDL and minimal impact of POPC sHDL, we investigated other sHDL-platelet interactions potentially affected by lipid composition. We first focused on cholesterol efflux abilities, which can be influenced by lipid changes (11, 12, 13). The lipids used in our sHDL formulation differ in length, transition temperature (T_m),_ and saturation, which affect their cholesterol affinity and binding properties (22, 23, 24).

**Fig. 3:**
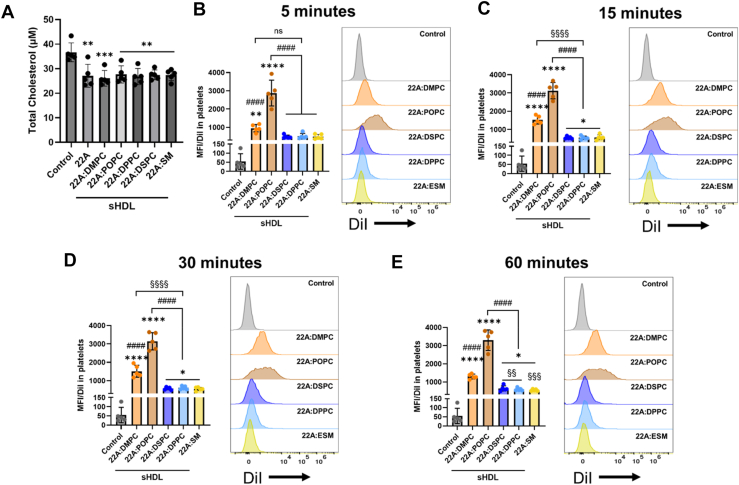
Differences in cholesterol removal capacity and cellular association of sHDL particles. (A) Washed human platelets were incubated with 22A peptide (100 μg/ml) and different sHDL particles (100 μg/ml of 22A peptide) for 15 min. The total cholesterol was quantified via Amplex Red Cholesterol Assay Kit. (B–E) Cellular associations of DiI-labeled sHDL particles (sHDLs at 100 μg/ml 22A, 200 μg/ml lipid, and 1 μg/ml DiI) following 5, 15, 30, and 60 min incubations with human washed platelets. Mean fluorescence intensity and representative histogram of DiI in platelets. Data represent mean ± SD (n = 5); ns = not significant, *∗P* < 0.05, *∗∗P* < 0.01, *∗∗∗P* < 0.001, *∗∗∗∗P* < 0.0001 versus control; ^*####*^*P* < 0.0001 versus 22A:POPC; ^*§§*^*P* < 0.01, ^*§§§*^*P* < 0.001, ^*§§§§*^*P* < 0.0001 versus 22A:DMPC.

To assess the impact of phospholipid composition on cholesterol efflux capacity, we incubated washed human platelets with sHDL nanoparticles and free 22A peptide at a concentration of 100 μg/ml. The total cholesterol was determined using the Amplex Red Cholesterol Assay. Our results showed that all sHDL formulations significantly decreased total platelet cholesterol (Fig. 3A). Cholesterol removal ranged from 22% to 26.5% across different sHDL compositions. Free 22A peptide demonstrated comparable efficacy, reducing cholesterol by approximately 23.7%. Notably, no statistically significant differences in cholesterol removal were observed between sHDL formulations or free 22A peptide.

We have previously shown that sHDLs associate with platelets, particularly those composed of DMPC (17, 18). Additionally, studies on lipid nanoparticles have demonstrated that cellular interaction is dependent on lipid composition (25, 26). To investigate the effect of lipid content on platelet associations with sHDL, we labeled sHDLs with DiI using the colyophilization method. Platelets were incubated with DiI-labeled sHDLs (sHDL at 100 μg/ml 22A, 200 μg/ml lipid, and 1 μg/ml DiI) for 5, 15, 30, and 60 min, and platelet-associated fluorescence was quantified via flow cytometry by measuring the mean fluorescence intensity of DiI. It should be noted that while we measure DiI signal associated with platelets, this methodology cannot definitively distinguish between complete particle internalization versus lipid transfer to the platelet membrane. The DiI signal indicates interaction between sHDL and platelets, but determining the precise nature and location of this interaction (surface binding, partial component transfer, or complete internalization) would require additional studies with different methodologies.

Our findings reveal distinct patterns at different time points. After a 5-min incubation, both DMPC and POPC sHDLs showed a significant DiI signal, with POPC sHDL demonstrating significantly greater association than DMPC (Fig. 3B). Following a 15-min incubation, platelets exhibited significant DiI fluorescence after incubation with DSPC, DPPC, and SM sHDLs (Fig. 3C). However, DMPC and POPC sHDL maintained higher levels of platelet-associated fluorescence compared to other formulations. At 30- and 60-min incubations (Fig. 3D and E), no significant changes in fluorescence intensity were observed, and all sHDL formulations maintained constant levels of platelet association. Notably, POPC sHDL exhibited the highest DiI fluorescence in platelets across all time points compared to other sHDL formulations.

### Inhibition of PLA_2_ diminishes the antiplatelet activity of DMPC sHDL

Despite POPC sHDL demonstrating higher platelet associations, DMPC sHDL exhibited superior antiplatelet activity. Notably, cholesterol efflux capacities remained comparable across all sHDL formulations, suggesting that that this property alone does not account for the observed differences in platelet inhibition. These findings, consistent with our previous observations (17), prompted us to explore alternative mechanisms by which DMPC sHDL specifically modulates platelet activity.

Therefore, we focused on the potential role of PLA_2_ enzymes, which are known to hydrolyze phospholipids found in lipoproteins, producing bioactive lipids that can modulate platelet function (27, 28, 29, 30, 31, 32, 33, 34, 35). To investigate whether PLA_2_ is involved in DMPC hydrolysis and subsequent antiplatelet activity, we examined multiple PLA_2_ enzymes found in platelets: cytosolic (cPLA_2_), secretory (sPLA_2_), calcium-independent PLA_2_ (iPLA_2_), and Lp-PLA_2_ (36, 37, 38, 39). We also included a phospholipase D (PLD) inhibitor to assess the specificity of DMPC hydrolysis to PLA_2_ activity. Platelets were incubated with specific inhibitors for each phospholipase: AACOCF3 (cPLA_2_), varespladib (sPLA_2_), bromoenol lactone (BEL, iPLA_2_), darapladib (Lp-PLA_2_), and FIPI (PLD) (36, 38, 40) before treatment with DMPC sHDL (100 μg/ml 22A peptide and 200 μg/ml DMPC).

**Fig. 4:**
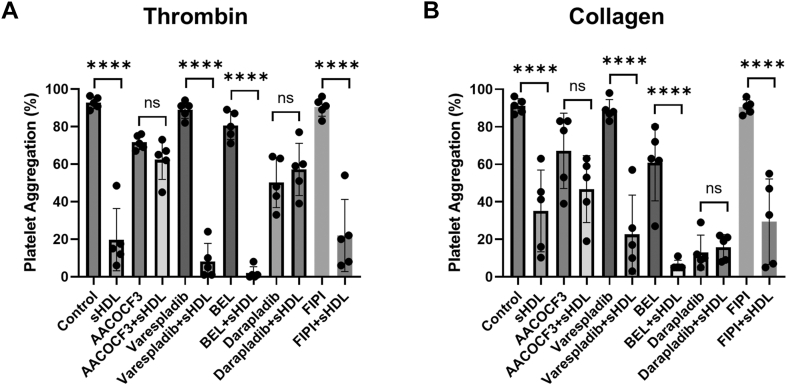
Inhibition of PLA_2_ diminished DMPC sHDL activity. Washed human platelets were incubated with phospholipase inhibitors: AACOCF3 (14 μM), varespladib (80 nM), BEL (3 μM), darapladib (10 μM), and FIPI (0.5 μM) for 10 min. Following incubation, platelets were treated with 22A:DMPC sHDL (100 μg/ml 22A and 200 μg/ml DMPC) for 15 min. Aggregation was induced with (A) thrombin (0.5 nM) or (B) collagen (2 μg/ml) and recorded for 10 min. Data is displayed as maximum aggregation as mean ± SD (n = 5); ns = not significant, *∗∗∗∗P* < 0.0001 versus controls.

Following thrombin stimulation (Fig. 4A), varespladib and BEL had no effect on sHDL activity, while AACOCF3 and darapladib significantly reduced sHDL’s ability to inhibit platelet aggregation. Similarly, with collagen stimulation (Fig. 4B), varespladib and BEL were ineffective, but AACOCF3 and darapladib inhibitors significantly blunted sHDL’s activity. We used a higher collagen concentration (2 μg/ml) to obtain measurable responses due to decreased activation following treatment with inhibitors. The observed decrease in aggregation, likely stems from PLA_2_ inhibition affecting the production of soluble agonists, particularly thromboxane A2 (TxA2), during platelet activation (30, 32, 33). Importantly, the PLD inhibitor FIPI did not affect sHDL’s capacity to modulate platelet activity with either agonist. These findings suggest that cPLA_2_ and Lp-PLA_2_ may contribute to the antiplatelet effects of DMPC sHDL, potentially through DMPC hydrolysis, although the involvement of additional PLA_2_ enzymes cannot be excluded.

Since DMPC is not a traditional substrate of Lp-PLA_2_, we performed enzymatic assays with recombinant human Lp-PLA_2_ to evaluate its ability to hydrolyze DMPC. While Lp-PLA_2_ demonstrated the capacity to hydrolyze DMPC, the activity was notably lower compared to its canonical substrates such as PAF C18, consistent with previous reports of minimal activity toward phospholipids with long-chain fatty acids (41) (Supplementary Fig. S2). Interestingly, DMPC in sHDL particles was hydrolyzed more efficiently than free DMPC, suggesting that the lipid environment in sHDL may facilitate enzyme access. Darapadib effectively inhibited Lp-PLA_2_-mediated hydrolysis of both free DMPC and DMPC in sHDL. These findings suggest a possible role for Lp-PLA_2_ in DMPC metabolism, although the relatively low hydrolysis activity indicates that other PLA_2_ enzymes may also contribute.

### Treatment with DMPC sHDL leads to production of PLA_2_ hydrolysis products in vitro and in vivo

Based on the observed substantial decrease in DMPC sHDL’s ability to reduce platelet aggregation following cPLA_2_ and Lp-PLA_2_ inhibitor treatment, we investigated whether DMPC undergoes PLA-mediated hydrolysis. PLA_2_ enzymes catalyze phospholipid hydrolysis at the *sn-2* position yielding an LPC and a free fatty acid (28, 29, 37, 38). For DMPC, this would yield LPC 14:0 and myristic acid. To detect these hydrolysis products, we incubated platelets with DMPC sHDL (100 μg/ml 22A peptide and 200 μg/ml DMPC) and analyzed both cell pellet and supernatant via LC-MS. We examined unstimulated and stimulated platelets to delineate specific PLA_2_ hydrolytic activity on DMPC.

**Fig. 5:**
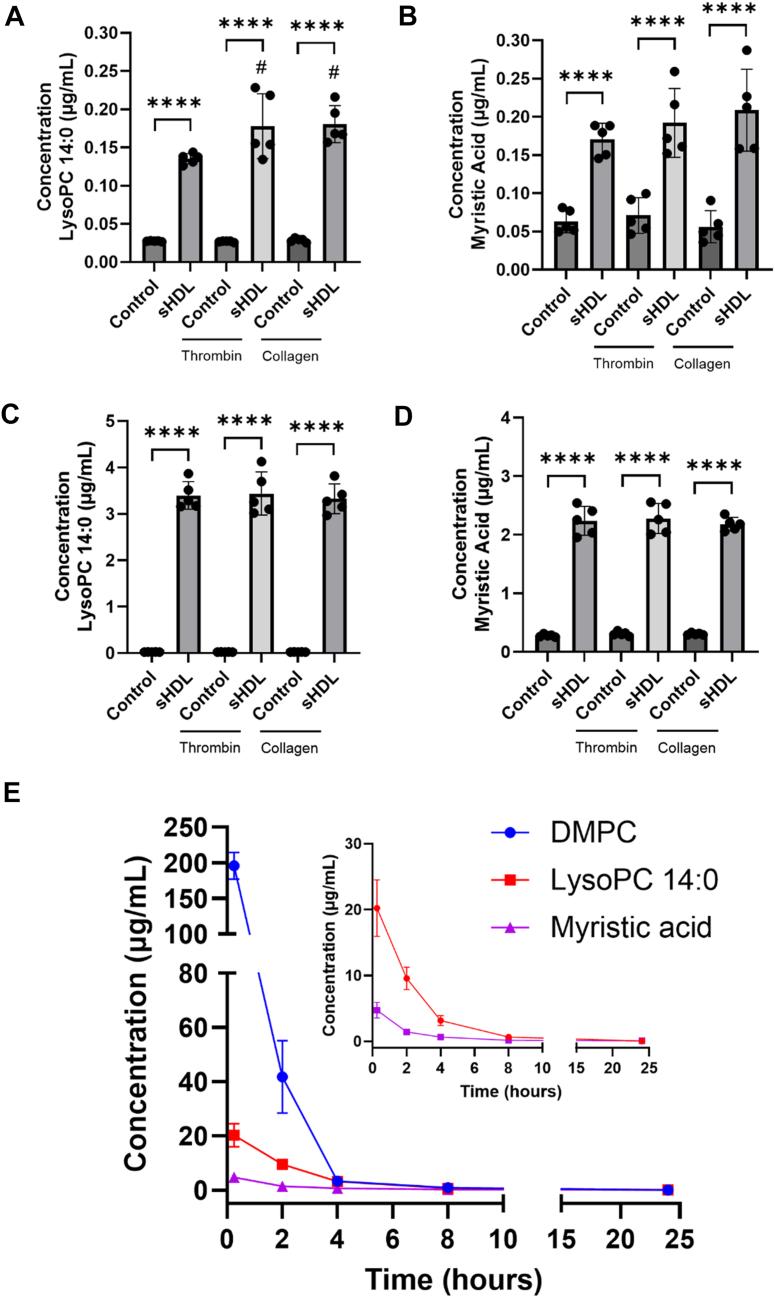
Analysis of DMPC-based sHDL PLA_2_ hydrolysis products in human platelets and in vivo. Washed human platelets preincubated with 22A:DMPC sHDL (100 μg/ml of 22A and 200 μg/ml DMPC) for 15 min and were activated with thrombin (0.5 nM) or collagen (1 μg/ml) for 5 min. Following treatments, the cell pellet and supernatant were collected for LC-MS analysis. Levels of LPC 14:0 and myristic acid, respectively, present in pellet (A–B) and in supernatant (C–D). Data represent mean ± SD (n = 5); *∗∗∗∗P* < 0.0001 versus control; ^*#*^*P* < 0.05 versus sHDL (unstimulated). (E) Mice were given a single intravenous injection of 22A:DMPC sHDL (100 mg/kg 22A and 200 mg/kg DMPC) and blood samples were collected at 0.25, 0.5, 1, 2, 4, 8, and 24 h after administration. Serum concentrations of DMPC, LPC 14:0, and myristic acid were determined by LC-MS. Data represent mean ± SD (n = 3).

Figure 5A and B show significant amounts of both LPC 14:0 and myristic acid in sHDL-treated platelet pellets. It is important to note that basal levels of myristic acid are seen since this fatty acid can be obtained through diet (42). Stimulation with thrombin or collagen slightly increased LPC 14:0 in sHDL-treated platelets, with significant increases for both agonists. The supernatant (Fig. 5C and D) contained higher levels of both LPC 14:0 and myristic acid, suggesting their secretion after production. Compared to controls, sHDL-treated platelets showed significant levels of LPC 14:0 and myristic acid. Once platelets were stimulated, sHDL treatment did not significantly produce more LPC 14:0 or myristic acid following stimulus.

To determine changes in DMPC levels, we quantified DMPC in platelets treated with sHDL. In the supernatant, DMPC concentration averaged 169.8 ± 18.1 μg/ml in unstimulated platelets, decreasing slightly to 157.3 ± 8.8 μg/ml and 153.1 ± 6.4 μg/ml of DMPC following thrombin and collagen stimulation, respectively (Supplementary Fig. S3). In the platelet pellet, DMPC levels were 2.7 ± 0.3 μg/ml prior to stimulation and modestly increased to 3.0 ± 0.3 μg/ml and 2.8 ± 0.2 μg/ml after thrombin and collagen treatment, respectively. Although these shifts were not statistically significant. The data suggests approximately 24% of the initial DMPC (∼223 μg/ml; Supplementary Table S1) was hydrolyzed under experimental conditions.

We previously showed that platelets treated with free DMPC did not inhibit aggregation (17), therefore we explored whether free DMPC would also become hydrolyzed by PLA_2_ in platelets. Free DMPC treatment significantly increased LPC 14:0 in both pellet and supernatant, but myristic acid levels were only significantly higher than controls in the supernatant (Supplementary Fig. S4). However, sHDL treatment led to significantly higher levels of both LPC 14:0 and myristic acid in pellet and supernatant compared to free DMPC treatment.

To determine whether other sHDLs with varying phospholipids produce hydrolysis products, we incubated unstimulated platelets with sHDLs containing POPC, DSPC, or DPPC. In the supernatant, POPC sHDL treatment resulted in significantly higher levels of LPC 16:0 and oleic acid compared to controls, while DSPC and DPPC sHDLs showed significantly elevated LPC 18:0 and LPC 16:0, respectively (Supplementary Fig. S5). No significant changes were observed in the corresponding fatty acids – stearic acid for DSPC and palmitic acid for DPPC – likely due to their high basal levels from dietary sources (43). In the platelet pellet, only POPC sHDL treatment led to significantly increased levels of its hydrolysis products, LPC 16:0 and oleic acid, whereas DSPC and DPPC treatments did not differ from controls.

To further validate these findings in vivo, we administered DMPC sHDL (100 mg/kg 22A peptide and 200 mg/kg DMPC) to mice and collected plasma at various time points (0.25, 2, 4, 8, and 24 h). LC-MS quantification of DMPC, LPC 14:0, and myristic acid revealed high DMPC levels 15 min post-injection, accompanied by increased LPC 14:0 and myristic acid (Fig. 5E and Supplementary Table S2). As DMPC concentrations decreased over time, LPC 14:0 and myristic acid levels also declined, plateauing after 8 h. At 24 h, DMPC was undetectable, but nanomolar concentrations of LPC 14:0 and myristic acid persisted. Control mice injected with vehicle exhibited expected small concentrations of myristic acid, but undetectable DMPC and negligible LPC 14:0 levels (Supplementary Table S2). These results provide evidence that DMPC in sHDL undergoes PLA_2_-mediated hydrolysis, producing LPC 14:0 and myristic acid both in vitro and in vivo.

### DMPC metabolites generated by PLA_2_ regulate platelet functions

Since we established that DMPC sHDL activity is dependent on PLA_2_, namely cPLA_2_ and Lp-PLA_2_, leading to production of metabolites, next we investigated whether these DMPC hydrolysis products directly modulate platelet functions. We evaluated how LPC 14:0 and myristic acid at different concentrations (1–50 μg/ml) influence platelet aggregation, integrin activation, and α-granule secretion.

**Fig. 6:**
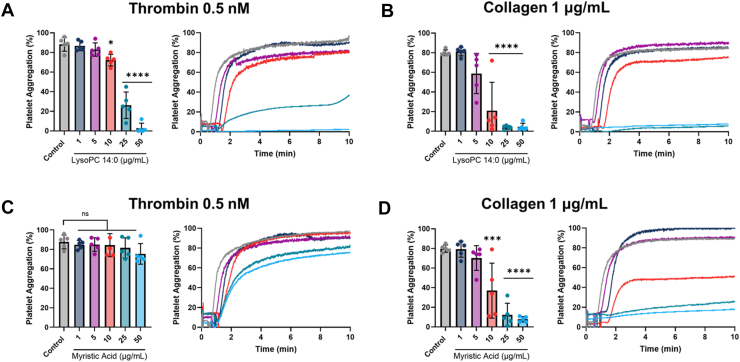
DMPC metabolites from PLA_2_ hydrolysis dose-dependently inhibit platelet aggregation. Washed human platelets were incubated with (A–B) LPC 14:0 or (C–D) myristic acid at concentrations of 1, 5, 10, 25, and 50 μg/ml for 10 min. Platelets were stimulated with thrombin (0.5 nM) or collagen (1 μg/ml) for 10 min. Data is displayed as maximum aggregation and representative traces, respectively. Data represent mean ± SD (n = 5); ns = not significant, *∗P* < 0.05, *∗∗∗P* < 0.001, *∗∗∗∗P* < 0.0001 versus control.

Platelet aggregation studies using thrombin (0.5 nM) and collagen (1 μg/ml) as agonists revealed distinct patterns for LPC 14:0 and myristic acid. LPC 14:0 exhibited clear dose-dependent inhibition for both agonists, with a minimum effective concentration of 10 μg/ml (Fig. 6A and B). In contrast, myristic acid only inhibited collagen-induced aggregation in a dose-dependent manner, also with a 10 μg/ml minimum effective concentration (Fig. 6C and D).

**Fig. 7:**
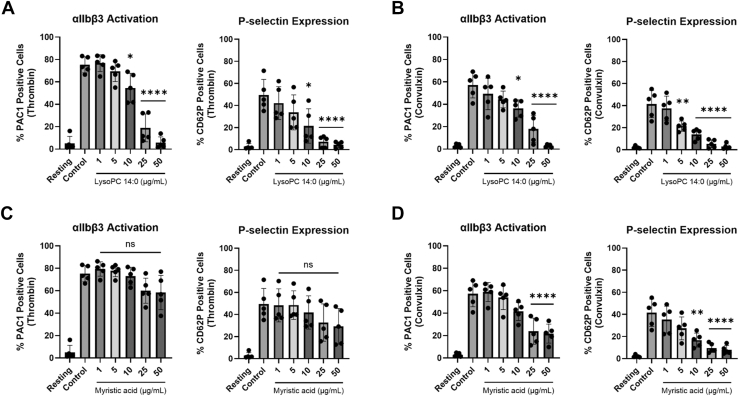
DMPC metabolites from PLA_2_ hydrolysis dose-dependently inhibit intracellular signaling. Washed human platelets were incubated with (A–B) LPC 14:0 or (C–D) myristic acid at concentrations of 1, 5, 10, 25, and 50 μg/ml for 10 min. Platelets were stimulated with thrombin (0.5 nM) or convulxin (25 ng/ml) in the presence of FITC-conjugated PAC-1 and APC-conjugated CD62P antibodies for 10 min and analyzed by flow cytometry. Flow cytometry data displayed as average of duplicate wells of % positive cells; αIIbβ3 activation displayed on left and P-selectin on right. Data represent mean ± SD (n = 5); ns = not significant, *∗P* < 0.05, *∗∗P* < 0.01, *∗∗∗∗P* < 0.0001 versus control.

We then explored the effects of these metabolites on key intracellular signaling markers via flow cytometry. We examined integrin αIIbβ3 activation and α-granule secretion in response to thrombin (0.5 nM) and convulxin (25 ng/ml), the latter being a snake venom utilized instead of collagen which directly activates the glycoprotein VI collagen receptor (44). LPC 14:0 caused dose-dependent inhibition of αIIbβ3 activation, indicated by reduced number of PAC-1 positive cells, for both thrombin and convulxin stimulation (Fig. 7A and B). It also inhibited α-granule secretion for both agonists, evidenced by reduced P-selectin expression or CD62P positive cells. LPC 14:0 was able to inhibit αIIbβ3 activation and α-granule secretion for concentrations above 5 μg/ml after thrombin stimulation, and significant inhibition was noted from 10 and 5 μg/ml, respectively, for convulxin-stimulated platelets.

Myristic acid exclusively inhibited αIIbβ3 activation and α-granule secretion dose-dependently following convulxin stimulation (Fig. 7D). The minimum concentration for αIIbβ3 activation inhibition was 25 μg/ml, while for α-granule secretion it was 10 μg/ml. Myristic acid showed no effect on thrombin-induced activation of these markers (Fig. 7C).

Since we show that other sHDLs lipid compositions led to the production of PLA_2_ hydrolysis products (Supplementary Fig. S5), we sought to determine whether the potency of these products influences the extent to which sHDL modulates platelet functions. To this end, we evaluated the capacity of these metabolites to inhibit platelet aggregation. We compared the inhibitory effects of these metabolites on platelet aggregation at both high (50 μg/ml; ∼107 μM for LPCs and ∼219 μM for fatty acids) and low (10 μg/ml; ∼21 μM for LPCs and ∼44 μM for fatty acids) molar concentrations relative to LPC 14:0 and myristic acid. At high concentrations, all LPCs exhibited similar efficacy in inhibiting aggregation for both thrombin and collagen stimulation (Supplementary Fig. S6). However, at low concentrations, LPC 14:0 was significantly more effective than LPC 16:0 in inhibiting aggregation induced by both agonists. Furthermore, myristic acid demonstrated superior inhibition of aggregation compared to stearic acid at high concentrations and outperformed both oleic and stearic acids at low concentrations following collagen stimulation. Notably, oleic acid was the only fatty acid to significantly inhibit thrombin-induced aggregation at high concentrations. These results show the unique potency of DMPC-derived metabolites and demonstrate the specificity of lipid composition in determining sHDL antiplatelet efficacy.

To further demonstrate that the DMPC hydrolysis products influence sHDL’s protective properties, we examined the combined impact of LPC 14:0 and myristic acid on platelet aggregation and activation markers. We utilized the metabolite levels from the supernatant we quantified in the previous section, which were approximately 3.4 μg/ml LPC 14:0 and 2.2 μg/ml myristic acid (Fig. 5C–D). The combined metabolites significantly inhibited platelet aggregation with both agonist (Supplementary Fig. S7A and B). In comparison to sHDL, the metabolites showed slightly less inhibition of aggregation, though this difference was not statistically significant. Similarly, the metabolites in combination significantly reduced integrin αIIbβ3 activation and α-granule secretion following thrombin and convulxin stimulation (Supplementary Fig. S7C and D). While sHDL exhibited a significantly greater capacity to inhibit integrin activation for both agonists, no significant difference was observed in P-selectin expression between the combined metabolites and sHDL. These findings indicate that PLA_2_-generated DMPC metabolites substantially contribute to the antiplatelet effects of DMPC sHDL.

## Discussion

The biological activities of both native and synthetic HDLs are profoundly influenced by their lipid composition (4, 10, 11, 12, 13, 14, 21, 45). Our previous research established that the type of lipid used in sHDL synthesis affects cholesterol efflux capabilities and anti-inflammatory properties (11, 12, 13, 14). We have demonstrated the antithrombotic activities of DMPC-based sHDLs both in vitro and in vivo (17, 18), revealing potent inhibition of various platelet functions through mechanisms not entirely dependent on cholesterol efflux but mediated by the CD36 receptor (17). Moreover, we have shown that this antiplatelet activity relies on both the protein and phospholipid components of sHDL. Expanding upon these findings, the current study investigates how lipid composition influences sHDL activity on platelets.

To investigate how lipid structural features influence sHDL’s effects on platelets, we selected lipids varying in chain lengths, saturations, and transition temperatures. First, we explored the ability of sHDL with varying lipids to inhibit platelet aggregation. Our results revealed that DMPC sHDL exhibited superior inhibition of aggregation for both thrombin and collagen agonists, while POPC sHDL was only effective at inhibiting thrombin-induced aggregation at lower concentrations (Fig. 2). Other formulations (DSPC, DPPC, and SM) showed no significant effects. We then confirmed that DMPC sHDL inhibits platelet aggregation in PRP (Supplementary Figure S1), which demonstrates that it can retain its potent antiplatelet activity in plasma. To further determine the lipid compositional changes on other aspects of sHDL function, we investigated the ability to remove total cholesterol. Since the efficacy of this process is largely determined by the characteristics of HDL’s constituent lipids (4, 22, 23, 24). Our results showed that all sHDL formulations, regardless of the lipid composition, exhibited similar and significant reductions in total cholesterol levels (Fig. 3). These reductions were comparable to those achieved by free 22A peptide, as previously reported (17). In platelets, cholesterol efflux by sHDLs appears to be primarily driven by the ApoA1 mimetic peptide rather than the lipid component. Notably, our prior study found that sHDLs consisting of both POPC and POPS had greater anti-inflammatory properties without increasing the cholesterol efflux abilities compared to POPC sHDLs (14).

These findings contrast with our previous results, which showed higher cholesterol efflux for POPC and DMPC-based sHDLs (11, 12), attributed to their lower T_m_ allowing these lipids to exit in a liquid crystal state at 37°C, thus facilitating cholesterol partitioning (11, 24). However, the discrepancy in results may be caused by several factors. Our previous experiments were conducted on macrophages, where sHDLs may interact differently compared to platelets. Furthermore, the incubation time differed significantly; our previous studies typically involved extended incubation periods (>4 h), whereas in our current study, platelets were incubated with sHDL for only 15 min, consistent with our efficacy studies. It is important to consider that platelets have a limited viable lifespan ex vivo, restricting the duration of incubation to maintain physiological relevance. Longer incubation times are not practical for platelets and could compromise data integrity. The fluorometric assay used here is well-established for quantifying cholesterol in platelets (46, 47, 48, 49) within these constraints. Taken together, differences in cell type and incubation time likely explain the observed discrepancies between studies.

Given our previous results of DMPC sHDL’s superior antiplatelet activity and the observed interaction between sHDL and platelets possibly involving the CD36 receptor (17, 18), we explored how lipid composition affects sHDL-platelet association. Despite comparable cholesterol efflux capacities, we observed marked variations in DiI fluorescence in platelets when incubated with different sHDLs (Fig. 3). Platelets incubated with POPC sHDL exhibited the highest level of fluorescence, followed by DMPC sHDL, while other sHDLs showed significantly lower association. Intriguingly, although POPC sHDL demonstrated greater platelet association than DMPC sHDL, its antiplatelet activity was considerably less potent. This discrepancy between the level of platelet association and antiplatelet efficacy suggests that the degree of sHDL-platelet interaction alone does not determine sHDL’s antiplatelet functions. These findings point to the involvement of additional mechanisms in sHDL’s activity, particularly DMPC-based formulations.

We next investigated the mechanisms underlying DMPC sHDL’s potent antiplatelet effects. We focused on PLA_2_ enzymes, known for their role in atherosclerosis pathophysiology and lipoprotein hydrolysis (27, 28, 29). PLA_2_ enzymes, namely Lp-PLA_2_ and sPLA_2_, hydrolyze phospholipids associated with lipoproteins to produce bioactive lipids such as LPCs that can contribute to atherogenesis (27, 28, 29, 30, 31, 32, 33, 36, 50, 51, 52). While Lp-PLA_2_ activity in LDL increases in disease states, its activity in HDL remains stable (27, 36). The ratio of HDL-Lp-PLA_2_ to LDL-Lp-PLA_2_ inversely correlates with disease severity, and increased Lp-PLA_2_ activity in HDL is associated with enhanced antioxidative properties (36, 45, 53, 54, 55). These findings suggest a protective role for HDL-associated Lp-PLA_2_.

In platelets, PLA_2_ enzymes are integral to the activation cascade. cPLA_2_ primarily catalyzes the production of arachidonic acid, which is subsequently metabolized into various bioactive lipids (37, 39, 56, 57, 58). Other types of PLA_2_ also contribute to platelet activation (39, 58). Notably, PLA_2_-generated products from oxidized LDL hydrolysis have been shown to activate platelets (32, 33, 59, 60, 61). On the contrary, previous studies show that sPLA_2_-modified HDL potently inhibits platelet functions due to LPC enrichment (34, 35). We hypothesized that DMPC sHDL’s enhanced antiplatelet activity might result from its interaction with PLA_2_, leading to the generation of bioactive lipid metabolites.

Our results revealed that the inhibition of PLA_2_ enzymes, particularly cPLA_2_ and Lp-PLA_2_, significantly reduced the antiplatelet effects of sHDL (Fig. 4). Although Lp-PLA_2_ is primarily involved in hydrolyzing platelet-activating factor and oxidized phospholipids, our enzymatic data indicates it can hydrolyze DMPC with relatively low efficiency (Supplementary Fig. S2). In addition, we observed increased hydrolysis of DMPC in sHDL particles by recombinant Lp-PLA_2_, suggesting that lipid organization influences enzyme accessibility. This phenomenon aligns with studies demonstrating that Lp-PLA_2_ tightly binds to DMPC vesicles, facilitating substrate access by adopting an open active site conformation that enhances enzymatic activity (41, 62, 63, 64, 65). Furthermore, the organization of DMPC in sHDL particles may further facilitate enzyme access. These factors may explain the inhibition of sHDL’s antiplatelet activity by darapladib, although previous research have demonstrated that darapladib exhibits off-target effects on different PLA_2_ enzymes (66). Therefore, the observed effects could be attributed to darapladib’s interaction with other PLA_2_ isoforms rather than solely Lp-PLA_2_.

We then confirmed the presence of PLA_2_ metabolites of DMPC in platelet incubated with sHDL. In unstimulated sHDL-treated platelets, we observed significant levels of LPC 14:0 and myristic acid present in both pellet and supernatant, with higher quantities in the supernatant (Fig. 5). This distribution suggests that these metabolites are produced by platelets and subsequently secreted, consistent with the known ability of platelets to release lipid mediators (67). We also confirmed that sHDLs containing other phospholipids (POPC, DSPC, and DPPC) generate PLA_2_ hydrolysis products, with higher levels of these products present in the supernatant compared to the pellet (Supplementary Fig. S5). Consistent with the degree of platelet association, POPC sHDL treatment resulted in higher metabolite levels in both supernatant and pellet compared to DSPC and DPPC sHDLs. These findings align with previous observations that lipoproteins, including HDL, can exchange lipids with platelets and alter the phospholipid composition of the membrane (68, 69), leading to increased levels of LPCs (33, 70).

The presence of DMPC metabolites in unstimulated platelets indicates the involvement of calcium-independent PLA_2_, primarily iPLA_2_ and Lp-PLA_2_ (28, 29, 52). Interestingly, the iPLA_2_-specific inhibitor, BEL, failed to reduce sHDL activity, indicating that this enzyme group may not significantly contribute to DMPC hydrolysis in platelets or may preferentially target other lipids. In contrast, we observed significant reduction in sHDL activity following Lp-PLA_2_ inhibition. Although, Lp-PLA_2_ is predominantly found in lipoproteins, platelets and other cells are known to secrete these enzymes upon activation (27). Notably, platelets contain two types of Lp-PLA_2_, with the most active form present in the cytosol (36). However, our enzymatic studies showed that Lp-PLA_2_ only minimally hydrolyzed DMPC compared to its preferred substrates (Supplementary Fig. S2). Therefore, as we mentioned previously, this could be due to effects on alternate PLA_2_ enzymes. Further studies are required to determine the contributions of specific PLA_2_ enzymes.

Platelet activation following incubation with sHDL resulted in minor increases in DMPC metabolite levels, despite the cPLA_2_-specific inhibitor AACOCF3 effectively diminishing sHDL’s activity. Given that cPLA_2_ is calcium-dependent and platelet activation increases intracellular calcium levels (28, 29, 37, 38, 39, 57, 58), we anticipated a more pronounced increase in metabolites. Several factors may explain this observation: 1) LPC 14:0 and myristic acid could be rapidly converted into other bioactive lipids upon platelet activation (71), 2) AACOCF3’s known inhibition of iPLA_2_ might contribute to the reduced sHDL activity in conjunction with its effects on cPLA_2_ (38, 72), and 3) the quantity of metabolites may be limited by the amount of DMPC that associates with platelets. The third option being particularly viable, as our observations of free DMPC treatment show production of metabolites, but at significantly lower levels compared to DMPC sHDL (Supplementary Fig. S4). Furthermore, quantification of DMPC in platelets treated with sHDL showed approximately 24% of DMPC was hydrolyzed during treatment. If PLA_2_ enzymes were solely responsible for this hydrolysis, we would expect at least ∼15 μg/ml of LPC 14:0 and myristic acid, which exceeds the measured levels. While PLA_2_ enzymes are the primary focus due to their established role in lipoprotein lipid hydrolysis and bioactive lipid production in platelets, other phospholipases may contribute to DMPC hydrolysis.

Our in vivo studies in mice provide compelling evidence for PLA_2_-mediated hydrolysis of DMPC sHDL, resulting in the production of bioactive lipid metabolites LPC 14:0 and myristic acid (Fig. 5). The persistence of these metabolites in plasma, even after DMPC becomes undetectable, suggests a prolonged antiplatelet effect of DMPC sHDL. These findings not only corroborate our in vitro observations but also highlight the potential of DMPC sHDL as a long-acting antiplatelet agent. It is possible that DMPC and its initial metabolites undergo further transformation into other lipids that contribute to the overall antithrombotic effects. It is important to note that our study focused primarily on PLA_2_-mediated hydrolysis, and other enzymes, such as LCAT, which also possesses PLA_2_ activity (4, 71), could potentially contribute to DMPC metabolism in vivo. A comprehensive lipidomic analysis should be conducted to elucidate the full spectrum of metabolites produced from DMPC, the PLA_2_ that are involved, as well as other phospholipases, and their respective contributions to the antiplatelet effects of sHDL.

We further demonstrate that DMPC metabolites, particularly LPC 14:0, can independently modulate platelet functions (Figs. 6 and 7). Both LPC 14:0 and myristic acid exhibited potent, dose-dependent inhibition of platelet aggregation and intracellular signaling markers. Given that sHDLs containing other phospholipids can also be hydrolyzed by PLA_2_ enzymes, we compared the inhibitory effects of their hydrolysis products on platelet aggregation to those of LPC 14:0 and myristic acid. At both high and low molar concentrations, LPC 14:0 was significantly more potent than LPC 16:0 in inhibiting aggregation induced by both agonists, while myristic acid was more effective than oleic and stearic acids at low concentrations following collagen stimulation (Supplementary Fig. S6). Notably, POPC sHDL produced higher levels of hydrolysis products (1.74 ± 0.14 μg/ml LPC 16:0 and 0.97 ± 0.43 μg/ml oleic acid) in the supernatant compared to DSPC (0.39 ± 0.09 μg/ml LPC 18:0) and DPPC sHDL (0.62 ± 0.08 μg/ml LPC 16:0), but not DMPC sHDL (3.40 ± 0.27 μg/ml LPC 16:0 and 2.24 ± 0.22 μg/ml myristic acid). This is interesting because POPC sHDL associates more strongly with platelets than DMPC sHDL yet generates fewer metabolites. This discrepancy may indicate that PLA_2_ enzymes have higher affinity for DMPC or that the lipid arrangement within sHDL particle facilitate better enzyme access to DMPC (73). This could further explain the relatively minor inhibition observed with POPC sHDL at low thrombin concentrations compared to the greater inhibitory capacity of DMPC sHDL. These results demonstrate that both the quantity and potency of hydrolysis products influence sHDL’s antiplatelet activity.

Building on the distinct inhibitory effects of individual metabolites, we next assess their combined influence at physiologically relevant concentrations. This approach allowed us to better approximate the overall contribution of PLA_2_-derived products to the antiplatelet activity of DMPC sHDL. The combination of LPC 14:0 and myristic acid significantly inhibited aggregation and activation markers, supporting their contribution to sHDL’s protective properties (Supplementary Fig. S7). Although these inhibitory effects were comparable to those of DMPC sHDL, they were slightly less pronounced, potentially reflecting additional mechanisms inherent to the particles, such as cholesterol efflux or direct receptor interactions mediated by DMPC, the 22A peptide, or both.

Notably, these metabolites alone required concentrations above 10 μg/ml to elicit significant inhibition, contrasting with the lower combined concentrations used here. However, achieving significant inhibition with the combined metabolites necessitated a longer incubation time (30 min) compared to 10 min for individual metabolites prior to stimulation. This difference likely arises from how these metabolites reach their targets: endogenously produced lipids act within localized microenvironments at higher effective concentrations, whereas exogenously added lipids must diffuse through the medium, requiring higher concentrations or extended exposure to produce comparable effects.

This differential potency and mechanism of action underscore the importance of specific lipid components of sHDLs for modulating platelet function. Similarly, lipid compositions influences native HDL functions, with previous studies demonstrating that HDL enriched in certain LPCs or fatty acids inhibits platelet aggregation (34). Research on various HDL fractions has revealed that HDL3c, the densest fraction with the highest LPC and PS content, possesses the greatest antithrombotic properties (10). Interestingly, HDL enriched in fatty acids 18:2 or 20:4 induced aggregation instead (34), highlighting the critical role of phospholipid composition in mediating the protective activity of native HDL. The protective properties of LPCs extend beyond platelets to various other cell types (71). For instance, LPC-rich HDL has demonstrated enhanced anti-inflammatory properties in neutrophils (74) and increased vasorelaxation and endothelial nitric oxide synthase activity in endothelial cells (55, 75).

This study illuminates the importance of lipid composition in shaping the antiplatelet effects of sHDL, in a similar fashion to native HDLs. Our findings unveil a novel mechanism by which DMPC-based sHDL exerts potent antiplatelet activity through PLA_2_-mediated hydrolysis, generating bioactive lipid metabolites LPC 14:0 and myristic acid. Although sHDLs containing other phospholipids also produce PLA_2_-derived products, these are generated in lower amounts and exhibit reduced potency compared to DMPC-derived metabolites. This specificity in both metabolite generation and functional impact underlies the superior efficacy of DMPC sHDL relative to other formulations. These insights advance our understanding of how sHDL composition influences antithrombotic function and underscore the potential for tailoring sHDL formulations to enhance therapeutic efficacy in thrombosis. Future studies should focus on identifying the specific PLA_2_ isoforms involved, conducting comprehensive lipidomic profiling to characterize the full spectrum of bioactive metabolites from various phospholipases, and exploring how these metabolites can be harnessed to further improve the antithrombotic properties of sHDL.

## Data availability

All data to support the findings in this study are contained within the manuscript and supplementary information.

## Supplemental data

This article contains supplemental data.

## Conflict of interest

M. H. is an equity holder and serves on the scientific advisory board for Veralox Therapeutics; is an equity holder and consultant for Cereno Scientific; and receives research funding from the NIH, Cereno Scientific, and Lexicon Pharmaceuticals. A. S. declares financial interests for board membership, as a paid consultant, for research funding, and/or as equity holder in EVOQ Therapeutics and ASKO Pharma. The University of Michigan has a financial interest in EVOQ Therapeutics, Inc. The remaining authors declare that they have no conflicts of interest.
