# Supplementary material for: Phospholipase A2 products influence the antiplatelet functions of synthetic high-density lipoproteins
Source: J Lipid Res. 2025 Dec 29;67(2):100972. doi: 10.1016/j.jlr.2025.100972 (PMC12857358; doi:10.1016/j.jlr.2025.100972)
Supplement: Supplementary — Material [file mmc1.docx]

| **Supplementary Table S1. Quantification of peptide and lipid in sHDL formulations (n=3, mean ± SD).** | | | |
| --- | --- | --- | --- |
| sHDL Formulation | Concentration (mg/mL) | | Ratio |
|  | 22A Peptide | Lipid |  |
| 22A:DMPC | 11.2 ± 0.2 | 22.3 ± 1.3 | 1:2.0 |
| 22A:POPC | 10.8 ± 0.3 | 19.8 ± 0.6 | 1:1.8 |
| 22A:DSPC | 9.9 ± 0.1 | 18.6 ± 1.4 | 1:1.9 |
| 22A:DPPC | 10.4 ± 0.7 | 22.4 ± 1.7 | 1:2.2 |
| 22A:SM | 11.4 ± 0.5 | 20.9 ± 1.1 | 1:1.8 |

**
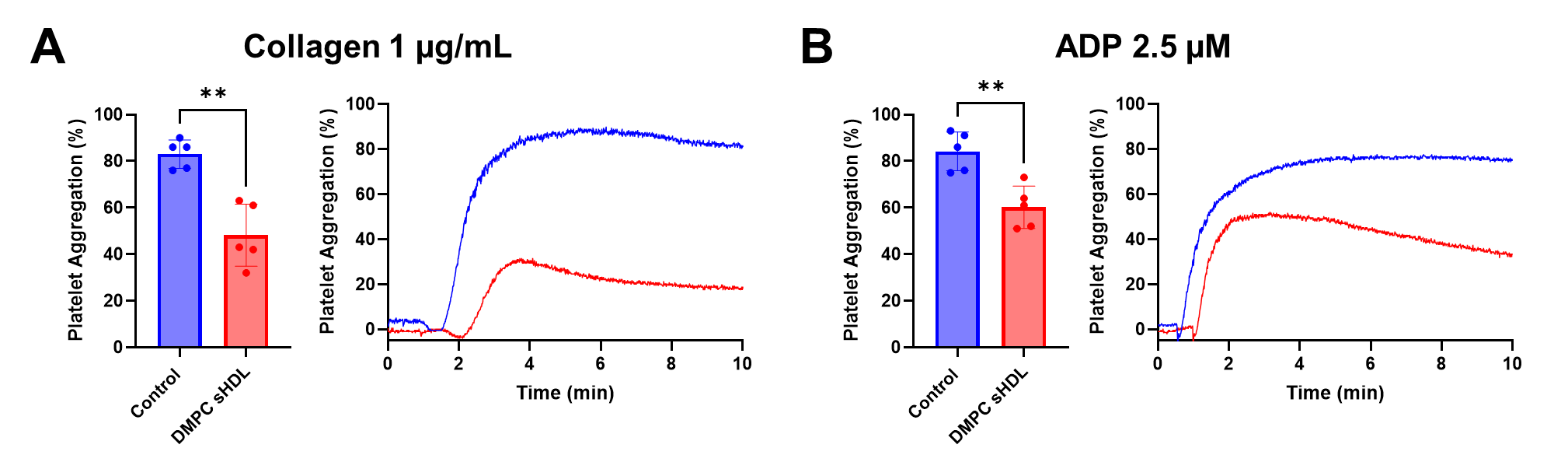
**

**Supplementary Figure S1. DMPC sHDL inhibits platelet aggregation in platelet-rich plasma.** PRP was incubated with DMPC sHDL (100 µg/mL of 22A peptide) for 15 min. Aggregation was induced with (A) collagen (1 µg/mL) or (B) ADP (2.5 µM) for 10 min. Data represent mean ± SD (n = 5), the maximum aggregation is displayed on the left and representative traces on the right; ***p*<0.01.


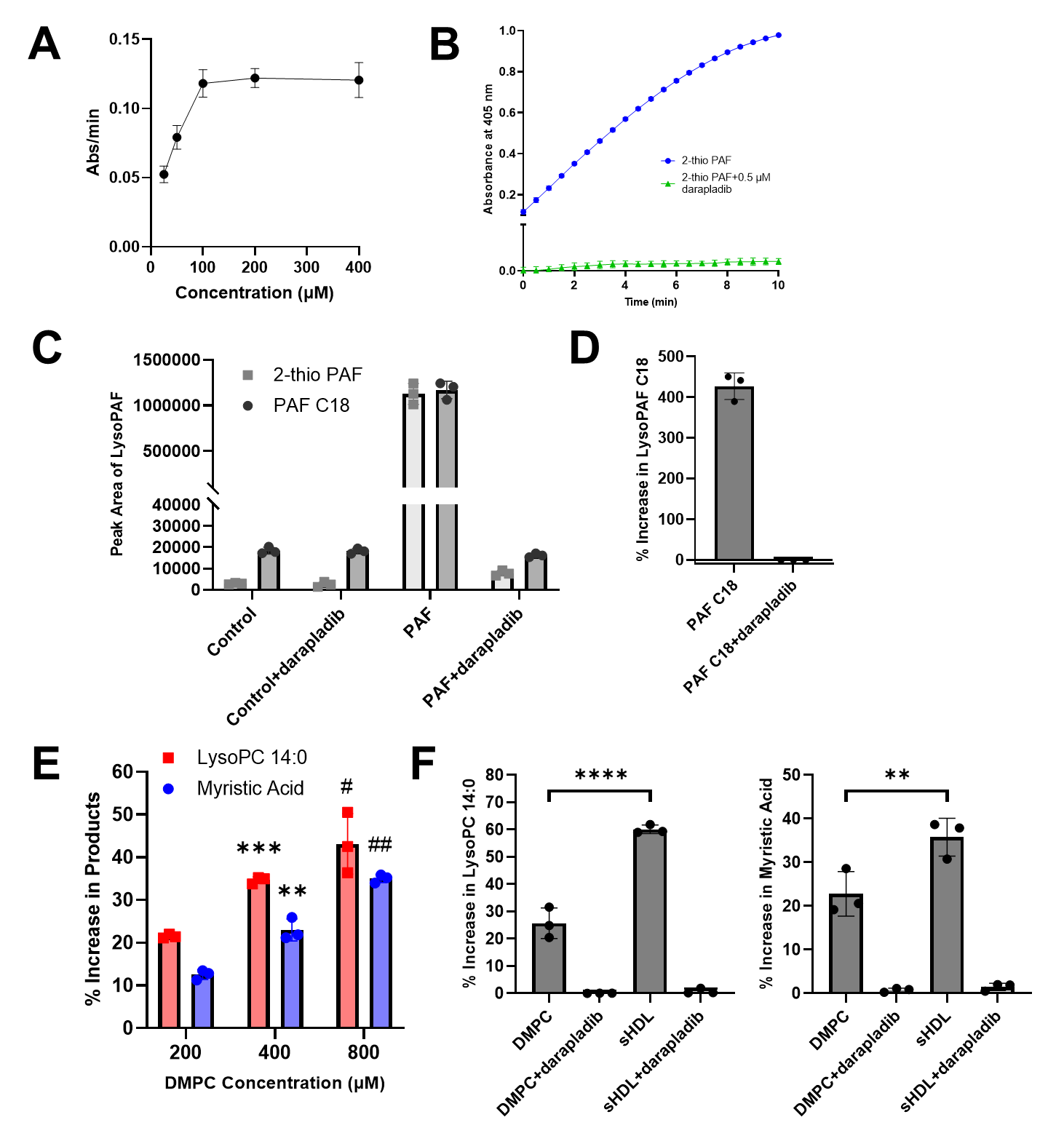


**Supplementary Figure S2. Recombinant human Lp-PLA_2_ enzymatic activity and hydrolysis of DMPC.** (A) Enzyme kinetics of recombinant Lp-PLA_2_ (2.5 µg/mL) using varying concentrations of 2-thio PAF substrate (25-400 µM) measured by colorimetric assay at 405 nm over 10 min. Initial velocity was calculated from the linear portion of the reaction curve. (B) Inhibition of Lp-PLA_2_ activity by darapladib (0.5 µM) assessed using 400 µM 2-thio PAF substrate. (D) LC-MS quantification of lysophospholipid products generated by Lp-PLA_2_ from 2-thio PAF and PAF C18 (400 µM) in the presence or absence of darapladib (0.5 µM). (D) Percentage increase of LysoPAF C18 in presence or absence of darapladib (0.5 µM) relative to control without enzyme. (E) Concentration-dependent hydrolysis of DMPC (200, 400, 800 µM) by recombinant Lp-PLA_2_ (2.5 µg/mL) measured by LC-MS quantification of LysoPC 14:0 and myristic acid production. (F) Comparison of hydrolysis between free DMPC and DMPC sHDL (both at 400 µM DMPC) by Lp-PLA_2_, with or without darapladib (0.5 µM). Data shows percentage increase of hydrolysis products relative to control without enzyme. Data are presented as mean ± SD from n = 3 independent experiments; ***p*<0.01, ****p*<0.001, *****p*<0.0001 vs. 200 µM DMPC concentration or as indicated; *^#^p*<0.05, *^##^p*<0.01 vs 400 µM DMPC concentration.

**
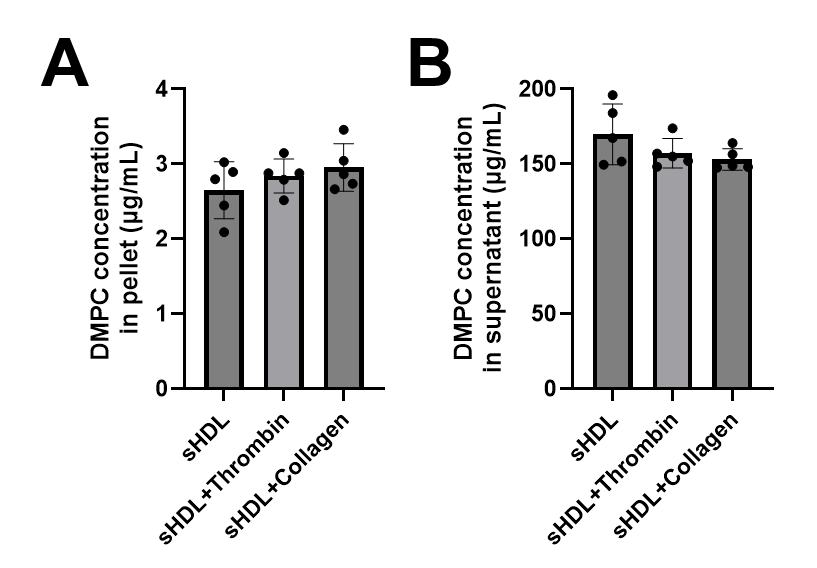
**

**Supplementary Figure S3. DMPC quantification in platelets incubated with sHDL.** Washed human platelets preincubated with 22A:DMPC sHDL (100 µg/mL peptide and 200 µg/mL DMPC) for 15 min. Platelets were stimulated with thrombin (0.5 nM) or collagen (1 µg/mL) for 5 min. The cell pellet and supernatant were collected for LC-MS analysis. Levels of DMPC present in pellet (A) and in supernatant (B). Data represent mean ± SD (n = 5).

**
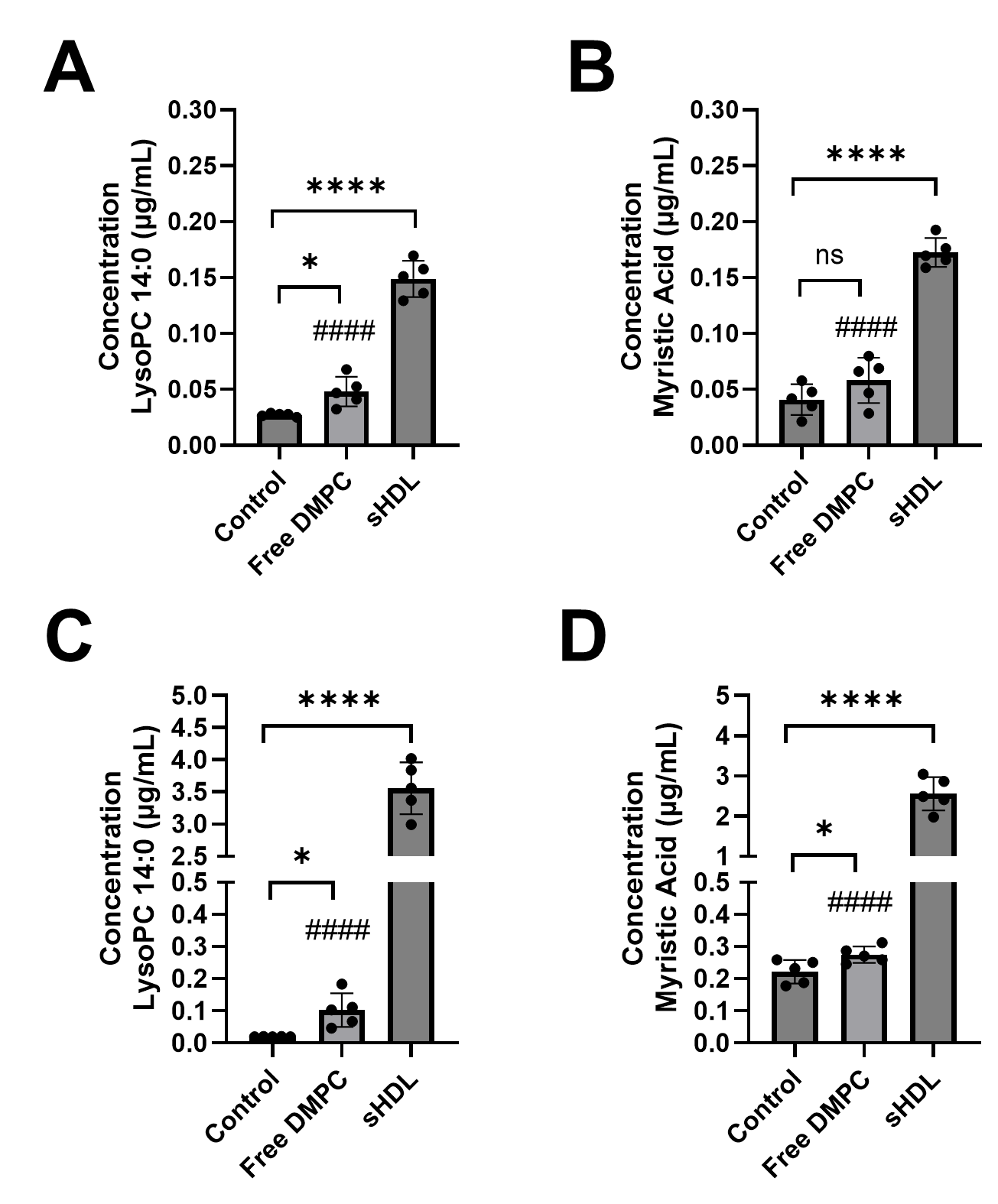
**

**Supplementary Figure S4. Quantification of PLA_2_ hydrolysis products in DMPC sHDL- and free DMPC-treated platelets.** Washed human platelets preincubated with 22A:DMPC sHDL (100 µg/mL peptide and 200 µg/mL DMPC) or free DMPC (200 µg/mL) for 15 min. The cell pellet and supernatant were collected for LC-MS analysis. Levels of LysoPC 14:0 and myristic acid, respectively, present in pellet (A-B) and in supernatant (C-D). Data represent mean ± SD (n = 5); ns = not significant, **p*<0.05, *****p*<0.0001 vs control; *^####^p*<0.0001 vs sHDL.


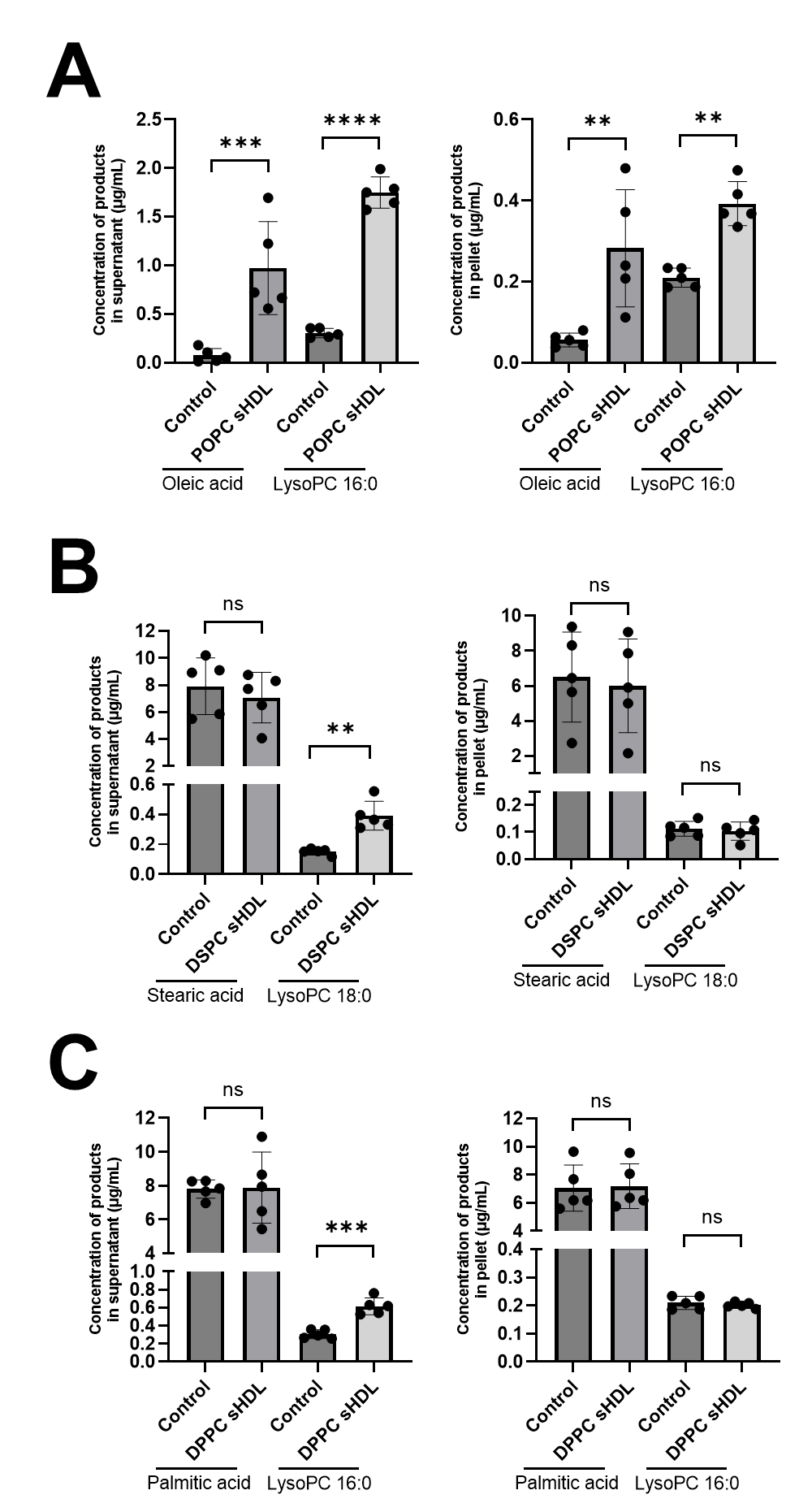


**Supplementary Figure S5. Quantification of PLA_2_ hydrolysis products in platelet treated with various sHDLs.** Washed human platelets preincubated with 22A:POPC sHDL, 22A:DSPC sHDL, and 22A:DPPC sHDL (100 µg/mL peptide and 200 µg/mL lipid) for 15 min. The cell pellet and supernatant were collected for LC-MS analysis. Levels of LysoPCs and fatty acids present in the supernatant (right) and pellet (left) are displayed for each sHDL. Data represent mean ± SD (n = 5); ns = not significant, ***p*<0.01, ****p*<0.001, *****p*<0.0001 vs control.

| **Supplementary Table S2. Quantification of DMPC hydrolysis by PLA_2_ *in vivo*. (n=3, mean ± SD).** | | | | |
| --- | --- | --- | --- | --- |
| **Treatment** | **Time point (hour)** | **Lipid Concentration (µg/mL)** | | |
|  |  | **DMPC** | **LysoPC 14:0** | **Myristic acid** |
| Control | 0.25 | - | 0.0056 ± 0.0032 | 0.062 ± 0.010 |
|  | 2 | - | 0.0034 ± 0.0030 | 0.073 ± 0.018 |
|  | 4 | - | 0.0024 ± 0.0010 | 0.051 ± 0.040 |
|  | 8 | - | 0.0003 ± 0.0023 | 0.077 ± 0.040 |
|  | 24 | - | 0.0052 ± 0.0010 | 0.018 ± 0.021 |
| DMPC sHDL | 0.25 | 195.70 ± 15.24 | 20.22 ± 3.50 | 4.72 ± 0.97 |
|  | 2 | 41.82 ± 10.96 | 9.54 ± 1.40 | 1.42 ± 0.11 |
|  | 4 | 3.24 ± 0.25 | 3.14 ± 0.63 | 0.64 ± 0.18 |
|  | 8 | 0.82 ± 0.37 | 0.65 ± 0.15 | 0.15 ± 0.09 |
|  | 24 | - | 0.057 ± 0.028 | 0.085 ± 0.031 |


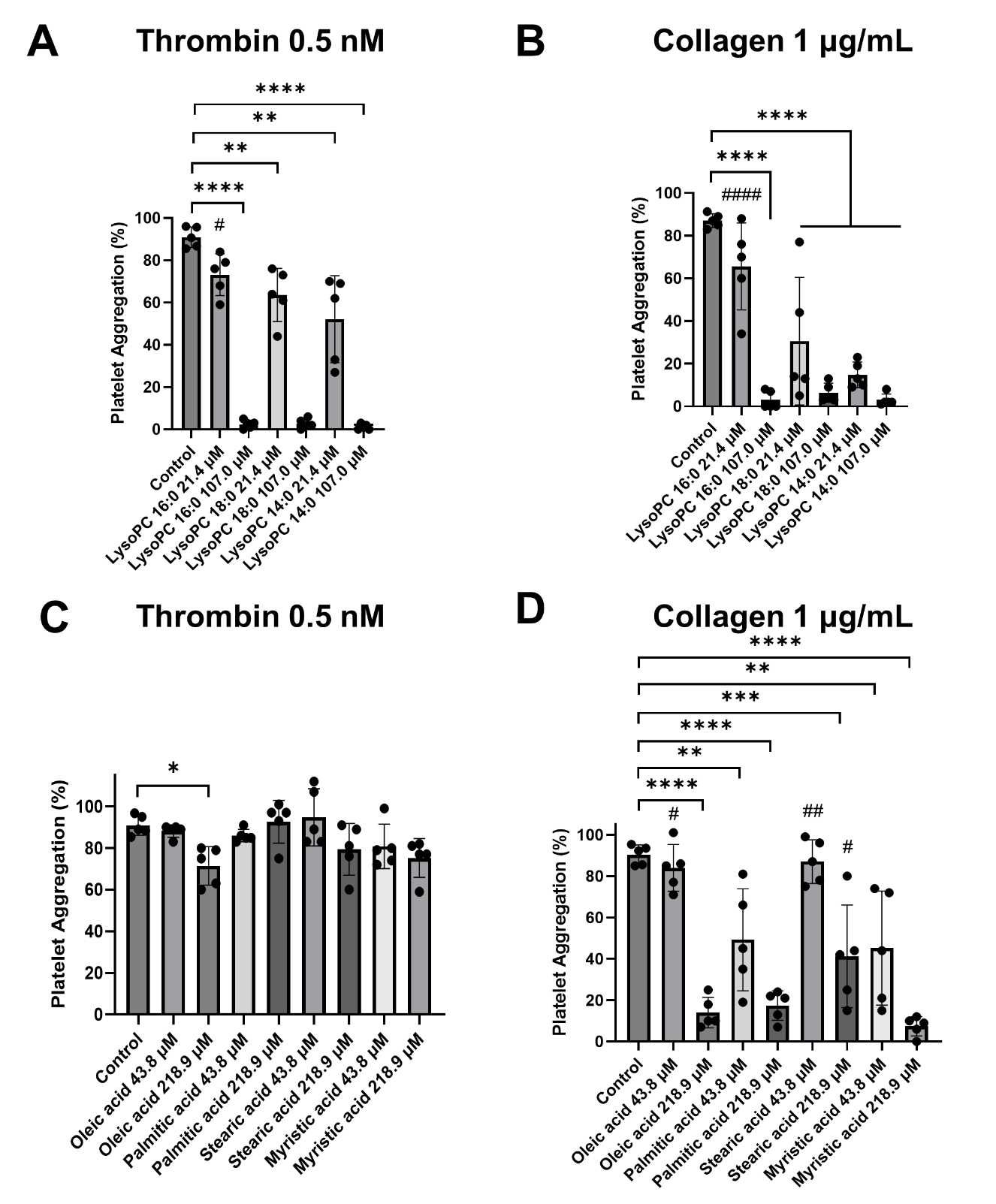


**Supplementary Figure S6. Differences in inhibition of platelet aggregation among PLA_2_ metabolites of sHDLs with varying lipids.** Washed human platelets were incubated with a high and low molar concentrations of LysoPC 16:0, LysoPC 18:0, LysoPC 14:0, oleic acid, palmitic acid, stearic acid, and myristic acid for 10 min. Platelets were stimulated with thrombin (0.5 nM) or collagen (1 µg/mL) for 10 min. Data is displayed as maximum aggregation. Data represent mean ± SD (n = 5); **p*<0.05, ***p*<0.01, ****p*<0.001, *****p*<0.0001 vs control, *^#^p*<0.05, *^##^p*<0.01, *^####^p*<0.0001 vs LysoPC 14:0 or myristic acid at equivalent concentrations.


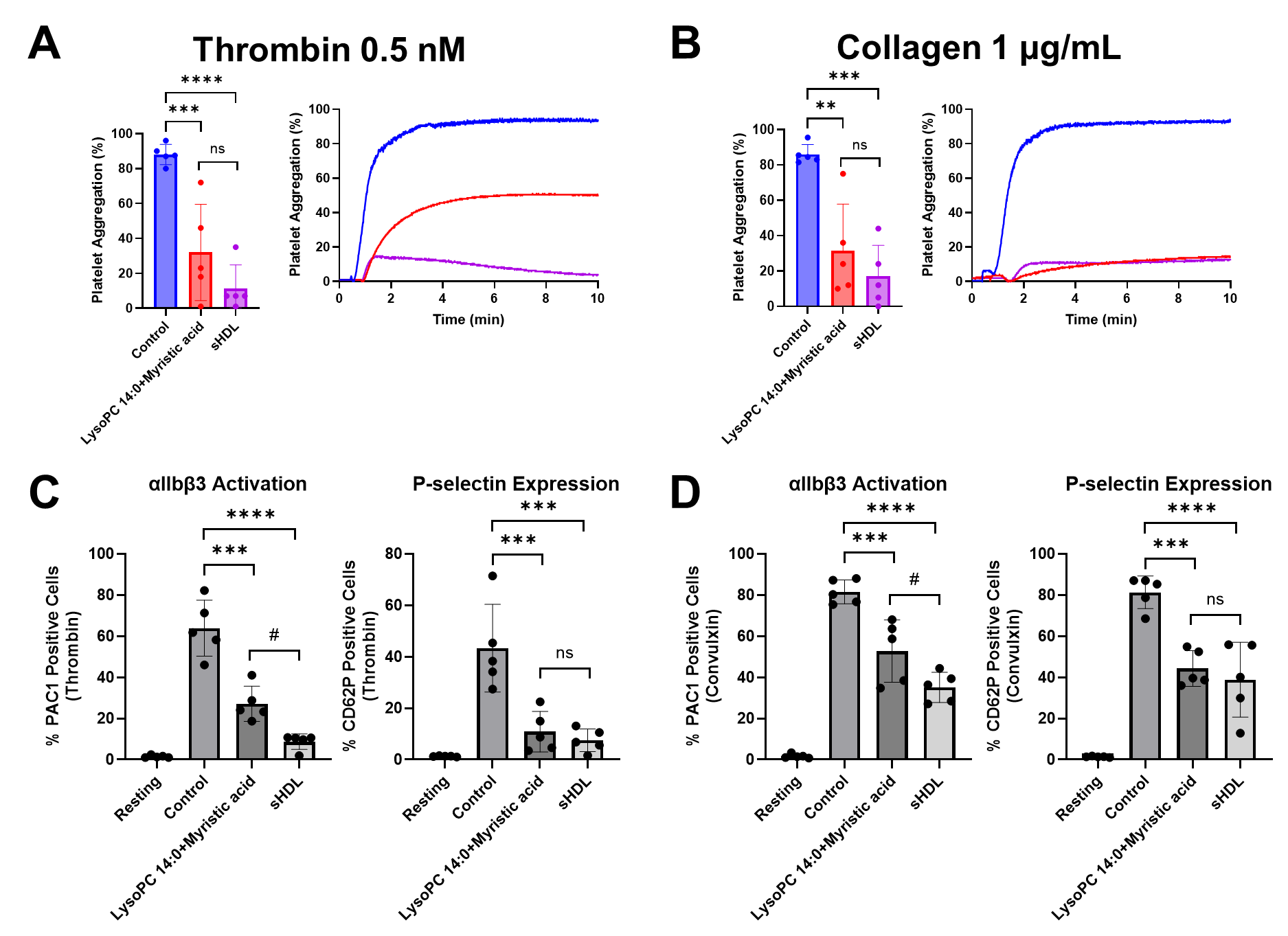


**Supplementary Figure S7. Combined effects of LysoPC 14:0 and myristic acid in comparison to DMPC sHDL.** Washed human platelets were incubated with both LysoPC 14:0 and myristic acid at concentrations 3.4 µg/mL and 2.2 µg/mL, respectively for 30 min or sHDL (100 µg/mL peptide and 200 µg/mL DMPC) for 15 min. (A-B) Following treatments, platelets were stimulated with thrombin (0.5 nM) or collagen (1 µg/mL) for 10 min. Data is displayed as maximum aggregation (left) and representative traces (right). (C-D) Activation markers measured via flow cytometry in platelets activated with either thrombin (0.5 nM) or convulxin (25 ng/mL) in presence of FITC-conjugated PAC-1 and APC-conjugated CD62P antibodies for 10 min. Flow cytometry data displayed as average of duplicate wells of % positive cells; αIIbβ3 activation displayed on left and P-selectin on right. Data represent mean ± SD (n = 5); ns = not significant, ***p*<0.01, ****p*<0.001, *****p*<0.0001 vs control, *^#^p*<0.05 vs sHDL.
